# Alternative set up strategies for upper tract robotic surgery with the Hugo RAS – experience from an early adopter institution

**DOI:** 10.1007/s11701-026-03563-7

**Published:** 2026-07-20

**Authors:** R. A. Keenan, R. Kelly, D. Moran, N. R. Bhatt, K. J. Breen, B. B. McGuire

**Affiliations:** 1 Department of Urology, St. Vincent’s Healthcare Group, Elm Park, Dublin 4, Ireland; 2 School of Medicine, University College Dublin, Dublin 4, Ireland; 3 Department of Surgical Affairs, Royal College of Surgeons in Ireland, Dublin 2, Ireland

**Keywords:** Robot-assisted surgery, Surgical ergonomics, Renal surgery, Hugo RAS

## Abstract

The Hugo RAS (Medtronic) represents a growing alternative to the DaVinci robotic platform, featuring a distinctive modular arm-cart design that may pose challenges for surgeons transitioning from established systems. Arm-cart positioning, restricted assistant access, and risk of personnel injury have been identified as barriers to adoption. Following FDA approval for urological procedures in 2025, optimizing a standardized, ergonomic set-up for upper tract cases is essential to facilitate broader uptake. A retrospective analysis of a prospectively maintained database was performed at a single robot-naïve institution. All patients undergoing Hugo RAS upper tract procedures — including robot-assisted partial nephrectomy (RAPN), radical nephrectomy, pyeloplasty, and nephroureterectomy — from January 2024 to May 2025 were included. Perioperative outcomes, set-up times, intraoperative events, and arm-cart repositioning requirements were recorded. A unilateral, four posterior arm-cart configuration with a linear or “W” port arrangement was employed in all. 54 upper tract procedures were performed across two expert robotic surgeons. No cases required intraoperative arm-cart repositioning. Median set-up time for RAPN improved by 8 min between the first and last quartile of cases (15 to 7 min). One additional port was required in a nephroureterectomy case. One minor assistant injury occurred. One case required open conversion for vascular bleeding. RENAL nephrometry scores ranged from 4 to 11 (median 7), with 44% classified as moderate-to-high complexity. A unilateral posterior arm-cart configuration with linear port placement is feasible, reproducible, and familiar to DaVinci-experienced surgeons, improving ergonomics, workflow, and set-up efficiency for Hugo RAS upper tract procedures.

## Introduction

The DaVinci robotic platform (Intuitive, Ca, USA) has been the market leader in robotic surgery since the early 2000’s when the first robotic cases were performed. There is worldwide familiarity with patient positioning, port placement as well as troubleshooting any issues such as arm clashing and working in a confined space. Particularly with the most recent Intuitive systems, the Xi, the DV5 and SP, there is flexibility with set-up and port placement as the arms originate from the same boom. Naturally, many challengers to the robotic surgery space have emerged with the Hugo RAS (robotic-assisted surgery) (Medtronic, MN, USA) gaining traction across Europe following CE approval in 2021 [1]. A key difference of this system is the multimodular approach to the arm carts which are individually placed around the operating table as well as an open surgeon console. This set up allows a highly customizable approach to arm-cart location and utilization with the option for both three and four arm approaches for many procedures such as renal or prostate surgery [2]. Difficulties in docking with arm clashing due to arm-cart positioning, restrictions on the bedside assistant as well as the potential for injury while confined due to arm-cart placement have been notable barriers for early adopters [3, 4]. Since the FDA (U.S food and drug administration) approval of the Hugo RAS for urological procedures in 2025, it could open this system to a market of over 200,000 robotic urology procedures in the U.S yearly [3]. A strategy therefore to maximise working space for the surgical assistant to avoid potential injury and optimize ergonomics while simplifying arm-cart placement and overall operating work flow is important to encourage ongoing utilization of the Hugo RAS and was recommended in a recent systematic review of Hugo RAS for robot-assisted partial nephrectomies [5]. We present an alternative and simplified strategy for arm-cart placement for upper tract robotic cases.

## Methods

A retrospective analysis of a prospectively maintained database on all patients undergoing surgery with the Hugo RAS system in our institution since the acquisition of the platform in early 2024 was performed. Currently, only urological procedures are being performed in our institution. Patients undergoing upper tract procedures such as robot-assisted partial nephrectomy (RAPN), robot-assisted radical nephrectomy (RARN) robot-assisted pyeloplasty (RAP) or robot-assisted radical nephroureterectomy (RANU) were selected for sub analysis. Baseline patient demographics, perioperative timings such as set-up time (defined as time from insertion of Veress needle to establish pneumoperitoneum to start of console time) as well as operative time (start of console to completion of operation) were recorded and analysed. Other characteristics such as side of operation, RENAL nephrometry score for those undergoing RAPN as well as intraoperative events related to arm cart position such as insertion of additional ports or repositioning of arm carts due to clashing or inaccessibility were recorded. The cases were performed in a robot naïve centre with staff inexperienced in robotic surgery however the operating surgeons are both expert fellowship-trained robotic partial nephrectomists with combined > 500 independent cases on the DaVinci system.

**Fig. 1:**
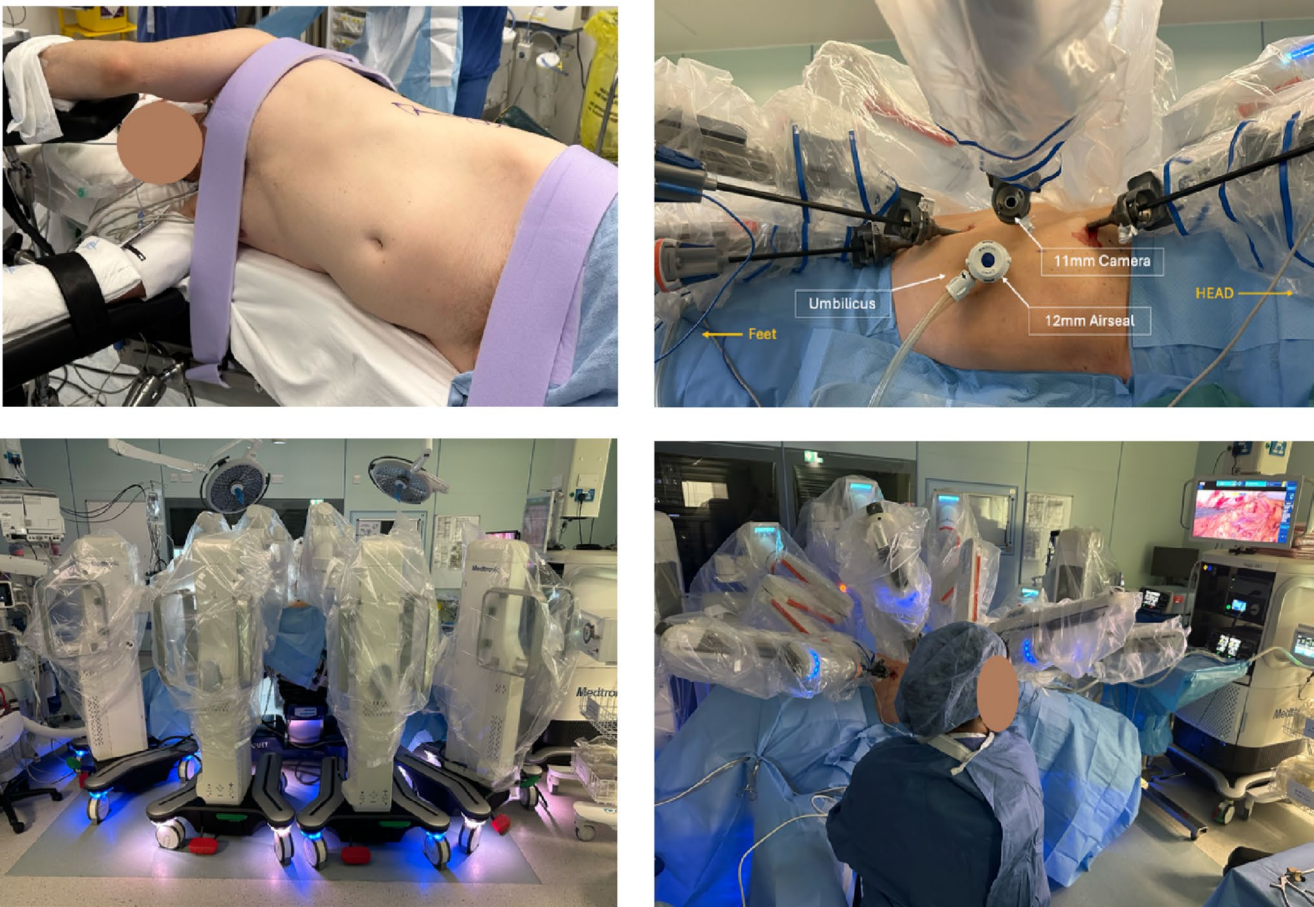
1a (top left) - standard positioning for kidney surgery. 1b (top right) – standard right side linear port alignment with Airseal device. 1c (bottom left) - arm-cart set-up as seen from behind. 1 d (bottom right) - arm-cart set up from assistant’s perspective

Our standard patient positioning for upper tract cases is to have the patient in the lateral position over maximal mid-table break at the approximate level of the umbilicus as shown in Fig. 1a. Pneumoperitoneum set between 12-15mmHg is established via a Veress needle in the approximate region of Palmer’s point for left sided cases and the mirror location for right sided cases. Port locations are marked out with the cranial-most port approximately 2 cm below the costal margin with subsequent ports approximately 8 cm apart. A linear approach, which we use as standard, as shown in Fig. 1b, can be used if space permits, or staggered in a “W” configuration across different geometric planes in cases where space across the abdomen is more limited [6]. An 11 mm visible entry port is then inserted to facilitate endoscope use with three further 8 mm ports inserted under vision as well as a 12 mm assistant port. Depending on surgeon preference, this may be an Airseal (Conmed, NY) 12 mm port (Fig. 1b) or else a standard 12 mm laparoscopic port with a further 5 mm Airseal port inserted near the anterior superior iliac spine (ASIS) to maintain pneumoperitoneum. Standard cart location is shown from behind in Fig. 1c and from the assistant’s angle in Fig. 1d. Figure two shows the standard angle and tilt values used. Angle variation is typically +/- 5^o^ between cases.

**Fig. 2:**
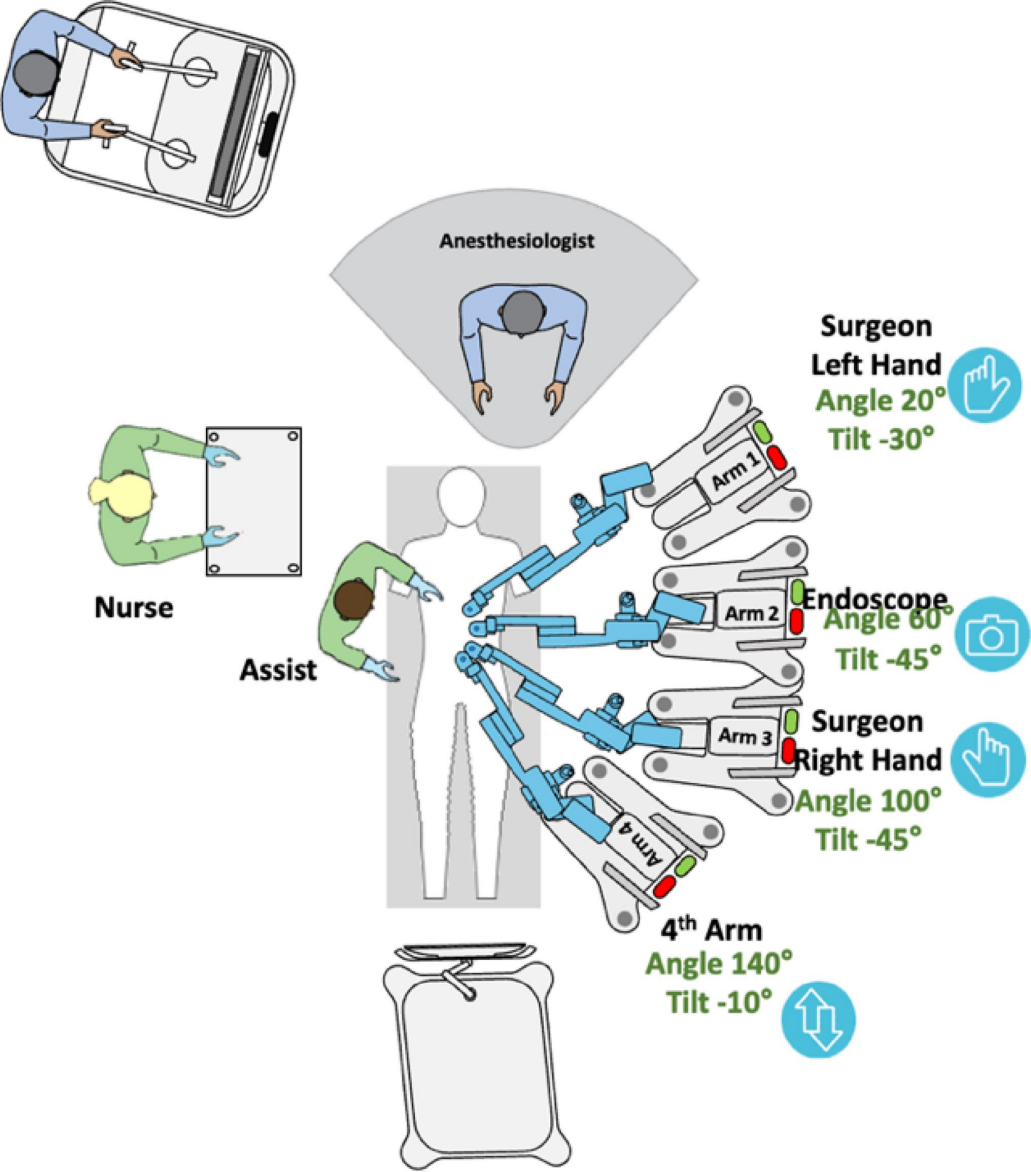
Standard unilateral Hugo RAS arm cart set up with relevant angles and tilts

## Results

Between the period of 1 st January 2024 and 1 st May 2025, 175 cases utilizing the Hugo RAS amongst three robotic surgeons were performed. Of these, 58 upper tract procedures were performed by two expert kidney surgeons. Excluding three retroperitoneal RAPNs which utilised a two-arm cart per side set-up, and the first upper tract case (RAPN) which utilised the recommended arm-cart set-up from Medtronic, all subsequent upper tract procedures (*n* = 54) utilised a unilateral arm-cart set-up as shown in figure one. Baseline demographics, perioperative characteristics and timings are shown in table one as well as RENAL nephrometry scores for partial nephrectomy cases and pathology results for any extirpative cases Fig. 2.

Specifically assessing the RAPN series of 36 cases as the largest component of the series, there was an improvement of 8 min in median set-up time (00:15 to 00:07) between the first and last quartile of cases. None of the 54 cases (0%) required intraoperative repositioning of an arm-cart once the operation had commenced. One case (RANU) (1.8%) required an additional 8 mm port to be inserted to allow reach to the distal ureter for the pelvic portion of the ureteric dissection however no uni-quadrant procedures required additional port insertion. Clashing between arms or instruments, although not formally recorded, was minimal and did not require any intraoperative arm or port readjustment during the study period. There was one occurrence of the bedside assistant being struck in the face by the left-handed arm-cart during a left sided procedure (minor injury, continued to assist for remained of case). One robot-assisted nephrectomy (1.8%) required emergency open conversion for uncontrolled renal vein bleeding Table 1.

**Table 1:** Baseline demographics and relevant timings of patients undergoing upper tract procedures utilising the Hugo RAS

Operation	*N*	Gender	Age (years)	Side	RENAL Nephrometry score	Path	Set up time	Operative time
RAPN	36	Male: 26 Female: 11	59 (IQR 54–69)	Left 20 (56%), Right 16 (44%)	7 (IQR 6–8.5)	pT1a – 21 pT1b – 6 pT3a – 1 Renal cyst – 2 AML – 3 Oncocytoma − 3	00:11 (IQR 00:05–00:24)	02:18 (IQR 01:55–02:46)
RAP	13	Male: 5 Female: 8	50 (IQR 39–63)	Left 6 (46%), Right 7 (54%)	N/A	N/A	00:11 (IQR 00:05–00:17)	01:37 (IQR 01:20–01:57)
RAN	4	Male: 3 Female: 1	69 (IQR 63–71)	Left 2 (50%) Right 2 (50%)	N/A	pT1b RCC – 2 Benign − 2	00:18 (IQR 00:11–00:22)	02:48 (IQR 02:15–02:58)
RANU	2	Male: 2	63 & 77	Left 1 (50%) Right 1 (50%)	N/A	pT3 UTUC − 2	00:13 (IQR 00:11–00:15)	02:51 (IQR 01:40–03:00)
Total	55	Male: 35 (60%) Female: 23 (40%)	N/A	Left 29 (53%) Right 26 (47%)	N/A	N/A	N/A	N/A

RAPN – robot-assisted partial nephrectomy. RAP – robot-assisted pyeloplasty. RAN – robot-assisted nephrectomy. RANU – robot-assisted nephroureterectomy. IQR – interquartile range. UTUC – upper tract urothelial carcinoma

## Discussion

The introduction of the Hugo RAS to clinical practice represents one of the main alternatives and competitors in the robot surgery landscape to DaVinci. A table highlighting key differences between the two platforms are shown in table two. Most robotic surgeons worldwide will be familiar with the set-up for ports, arm positions as well as troubleshooting intraoperative issues related to clashing, therefore newer consoles are likely to pose certain challenges in this regard which may dissuade institutions from acquiring such systems, particularly in the case of transitioning to a modular arm-cart set-up. Here we have described our port set-up as well as arm cart placement which is an alternative to the standard recommended set-up by Medtronic and an initial case series for renal surgery [6, 7]. Our standard linear port set-up is analogous to the linear set-up used by the DaVinci Xi system and has been utilised in all our renal cases bar four, highlighting ease of transferability across different robotic systems. An alternative port set-up where abdominal working space is limited (short abdomen, short or small patient etc.) is to stagger ports in the “W” configuration as well as to place them in different geometric planes allows surgeon flexibility to choose a bespoke patient set-up Table 2.

**Table 2:** Comparative table of Hugo RAS vs. DaVinci robotic platforms

Feature	Hugo RAS (Medtronic)	da Vinci (Intuitive Surgical)
Arm architecture	4 independent arms on separate mobile towers; each arm mounted individually	Single patient-side cart with 3–4 arms on a central boom (Si/Xi/SP variants)
Port placement flexibility	High — arms placed independently, ports can be positioned more freely to suit anatomy	Moderate to High: port placement more constrained by fixed boom geometry
Docking/setup time	Longer — each arm docked separately; currently a recognised limitation in early-adopter data	Xi/SP fast: highly drilled teams achieve < 10 min; single-cart docking is quicker
Assistant working space	Variable — independent arm spacing can create more room for a bedside assistant to work between arms but can also constrain if bilateral arm-cart placement	Comfortable – less floor space utilised by single boom
External arm collision	Higher theoretical risk given independent arm freedom; requires careful port planning and assistant spatial awareness	Lower with Xi due to boom geometry managing inter-arm spacing; SP near-eliminates it for single-port
Multi-quadrant access	Good — arms can be repositioned independently without full redocking	Xi designed for multi-quadrant; SP may be limited if contralateral access required
OR footprint	Larger overall — multiple towers occupy more floor space	Xi/SP compact single-cart design; smaller overall footprint than Hugo’s multi-tower setup
Surgeon ergonomics	Open console; less immersive; surgeon posture not as optimised. Improved operating room dynamics	Closed immersive console with superior 3D visualisation and ergonomic seating; well-established
Haptic feedback	None currently	Only DV5
Instrumentation	Growing portfolio; wristed instruments available; some gaps vs. da Vinci breadth	Extensive mature portfolio across all specialties; EndoWrist instruments with 7 degrees of freedom
Cost	Lower capital cost; potentially more competitive pricing; useful in cost-sensitive or public health systems	Higher capital and per-procedure cost; dominant market position means less price flexibility
Availability/installed base	Limited — newer platform, fewer centres, less global service infrastructure	Very large global installed base; mature service/support network; extensive training ecosystem
Evidence base	Growing but limited; mostly early feasibility and comparative case series	Extensive — decades of published data across urology, gynaecology, colorectal, thoracic, and HPB
Regulatory status	CE marked; FDA cleared for select procedures; expanding indications	Broadly cleared across a wide range of procedures globally

CE – Conformitè Europèenne. FDA – U.S Food and Drug Administration. HPB – Hepatopancreaticobiliary

**Fig. 3:**
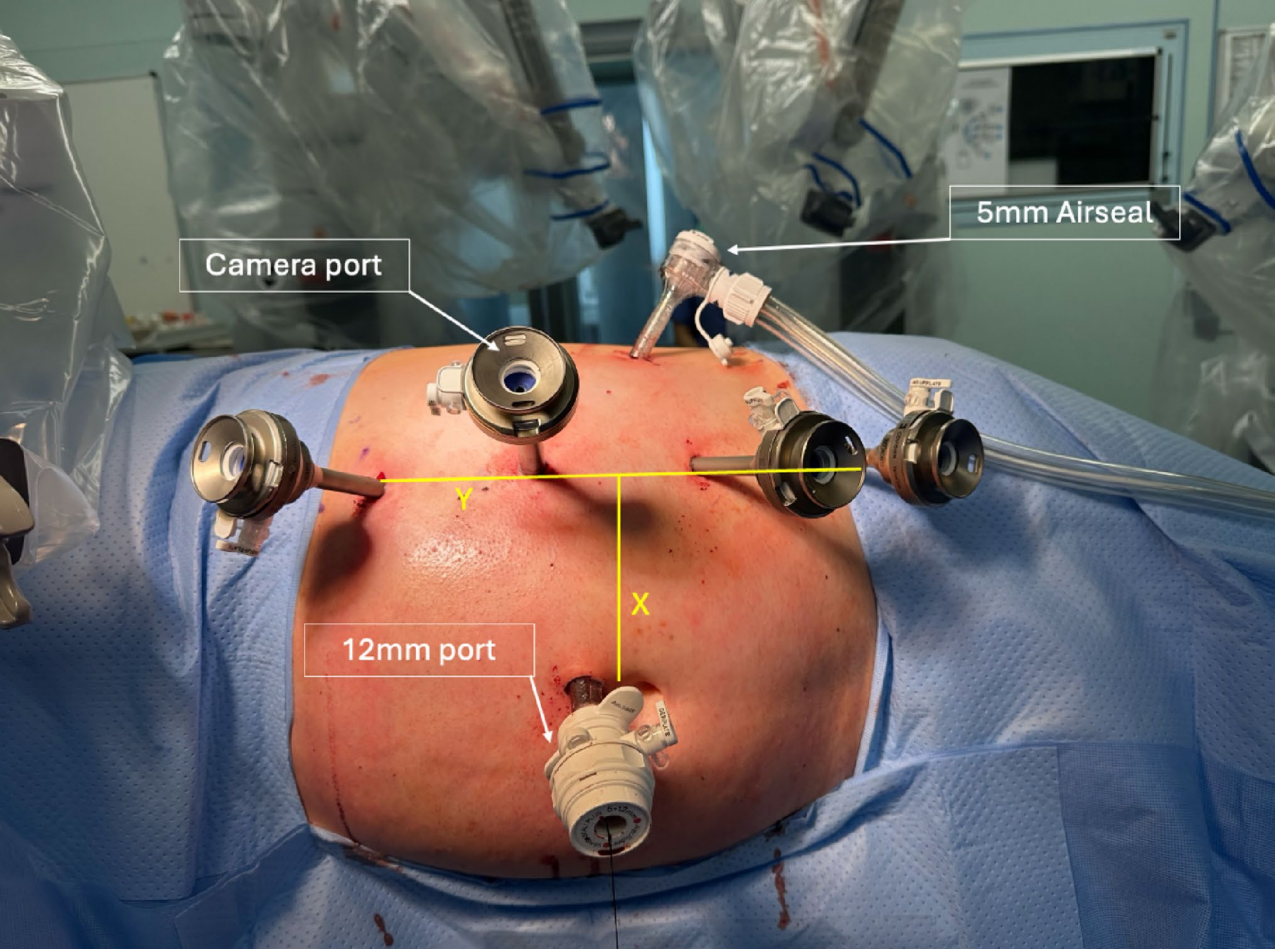
Linear port alignment for left kidney operation with additional 12 mm assistant port and 5 mm Airseal port

Based on our experience with our set-up, some key points for success should be noted. Maximising the space between individual ports is important to avoid clashing therefore a minimum distance of 8 cm should be prioritized. Secondly, the assistant port should ideally be placed in a vertical plane (line X, Fig. 3) perpendicular the midpoint of a line drawn between arms 2 and 3 (line Y, Fig. 3) to allow space for the assistant to access the port for suctioning, clip application etc. Furthermore, the distance between the assistant port and the linear port line (distance of line X as shown in Fig. 3) should be maximized as much as possible by placing the ports as laterally as is achievable from the midline. Intraoperatively, this mean the operative arms are less likely to clash with the assistant and permit more access for the assistant, optimizing their ergonomics. These features are highlighted in figure three.

Early series of pyeloplasty reported a median docking time of between 4 and 8 min although one of these series of five patients only utilised three robotic arm-carts [8, 9]. Studies reporting on docking time for partial nephrectomy show quite a wide variation from under 4 min to over 20 min with one study reporting a statistically significant longer docking time of 20 min with the Hugo vs. 12.5 min with the DaVinci platform [10–12]. A large multicenter RAPN series using the Hugo RAS reported a median docking time of 5 min with operative time of 79 min while showing a statistically significant effect on progressive case volume and lower docking times therefore efficiency with docking the robot arm-carts appears to be related to the experience and comfort of the surgeon using the system [13]. In our data, the trendline of set-up time vs. time showed an R^2^ = 0.0123 which would imply no statistically significant improvement over time however when broken down into quartiles, a decrease of 8 min in set-up time represents a pragmatic improvement. As early as the 4th case in our RAPN series, set-up time was as short as 5 min whereas the 32nd case took 24 min reflecting real world situations of an unselected case series where surgeons may encounter the obese patient, variations in abdominal and torso size, prior incisions, taking down adhesions etc. Larger multi-institutional or metanalytical studies are likely to be required to actually assess if there is a real difference in set-up times between the different platforms although the authors would argue that comfort and familiarity with the nuances of set-up are more practically relevant than reducing set-up time.

Alternatives to the four-arm set-up can also be considered. As discussed earlier, Hugo RAS pyeloplasty has been performed using three robotic arms only. The approach of using only three robotic arms as well as two 12 mm assistant ports has also been described and used with high success rates in RAPN including in moderate to high complexity renal masses, plus an off-clamp series [14, 15]. Notably in our series, we describe a range of RENAL nephrometry scores ranging from four to eleven with a median of 7 and 44% of cases being of moderate or high complexity (RENAL nephrometry of 7 or greater), highlighting how our approach works in a consecutive series of unselected complexity mix. One patient had a pathological positive margin (2.7%). These are in keeping with contemporary series of partial nephrectomy’s performed with the Hugo RAS (15).

While there have been no documented injuries to bedside assistants for Hugo RAS cases apart from this paper, it is well recognized that there is reduced working space for the surgical assistant and collisions with personnel have likely gone unreported. We advocate that our proposed streamlined set-up frees up a considerable amount of space for the assistant to utilise and allows them to be positioned to avoid arm collision. By combining other recommendations such as raising the operating table height by Bobrowski et al., risks to the assistant can be mitigated.

Two publications have described this arm-cart set-up in upper tract cases. In an Italian multicenter study, one of the five participating institutions utilised a similar four posterior arm-cart set up although it is unclear how many of the 140 patients were operated on with this set-up. One further study assessing the Hugo RAS in performing robot-assisted nephroureterectomies in five patients performed the first case with one arm-cart on the anterior side of the patient however encountered significant limitations in range of access therefore moved all arm-carts posteriorly while keeping a “W” style port set-up for the subsequent four cases without issue and recommended this set-up as standard [16]. Considering the multiquadrant access required to perform a nephroureterectomy, avoidance of clashing is paramount therefore without any evidence to the contrary, this appears to be the optimal set-up and is validated in our centre’s experience.

The benefit of this set-up also extends to operating room ergonomics and workflow, capitalizing on the open console design of the Hugo RAS which has already been shown to improve operating room dynamics and efficiency [17]. All the arm carts can be positioned on one side of the operating room allowing ease of access for the patient bed entering and leaving as well as transitioning the patient from the operating table without the need to maneuver the anteriorly positioned arm cart out of the way, particular when four separate arm carts take up more physical storage space compared a single-cart system [7]. Furthermore, the anteriorly located arm cart works off a much lower height compared to the posteriorly located arm-carts. Bearing in mind that it utilizes a negative docking angle, this can cause issues with limited range of motion necessitating excessively high operating table height or intraoperative compromised range of motion. By placing all four arm-carts posteriorly, this issue is negated.

Our paper has certain limitations. Body mass index (BMI) would have been a useful range of values to have to objectively demonstrate the various habitus types that can be accommodated with this approach. Similarly, we did not record the docking time separate from the overall set-up time which included establishing pneumoperitoneum therefore our reported times likely overcall the time required to insert ports and dock the individual arm carts. Although we did not need to reposition any arm-carts intraoperatively there were certainly instances when arms clashed resulting in alarm and disabling of the system. While these alerts were not recorded, they occurred occasionally and were not felt to have interfered with the flow of the case although are distracting for the surgeon. We did not report on a direct comparison of set-up times between DaVinci and Hugo RAS platforms from our institution. The Hugo RAS is utilised in the private hospital on the healthcare campus and used exclusively by consultant surgeons whereas the DaVinci is utilised by a mix of urological trainees and consultants, therefore, considering the impact of skill acquisition and training on set-up and operative times, these will naturally be longer. Therefore, a direct comparison was not felt by the authors to accurately reflect a true difference. Key features of our series however are that it represents an unselected patient cohort in terms of gender, height/weight/BMI, tumour burden or procedure and proves feasibility of the four posteriorly located arm-carts with either a linear or “W” port configuration for a range of renal procedures.

## Conclusion

The Hugo RAS differs significantly from the DaVinci system in terms of modular arm cart design which requires a different approach to port placement, arm-cart set-up and overall surgical approach which has the potential to disrupt the widespread uptake and adoption of a new robotic platform. Based on our experience of multiple upper tract procedures spanning a broad range of complexity, we propose an alternative surgical set-up grossly analogous to the linear set-up that robotic surgeons familiar with the DaVinci systems will be used to. We have shown that set-up time reduces with increased usage and outcomes remain consistently high despite taking on high complexity work. The Hugo RAS seems to pose more threat of injury to the bedside assistant however the ergonomics and workflow are considerably simplified with our proposed standardized set-up. We hope that by sharing an alternative approach to modular arm-cart surgery, it will reduce the burden of transitioning to newer robotic systems for experienced surgeons and encourage institutions to consider alternative platforms.

## Data Availability

All data supporting the findings of this study are available within the paper.
